# Association of Famine Exposure on the Changing Clinical Phenotypes of Primary Hyperparathyroidism in 20 years

**DOI:** 10.3389/fendo.2022.907019

**Published:** 2022-06-17

**Authors:** Tian-jiao Yuan, Yu-ying Yang, Min-ting Zhu, Yang He, Lin Zhao, Wen-zhong Zhou, Ting-wei Su, Hong-yan Zhao, Li-hao Sun, Bei Tao, Jian-min Liu

**Affiliations:** ^1^ Department of Endocrine and Metabolic Diseases, Shanghai Institute of Endocrine and Metabolic Diseases, Ruijin Hospital, Shanghai Jiao Tong University School of Medicine, Shanghai, China; ^2^ Shanghai National Clinical Research Center for Metabolic Diseases, Key Laboratory for Endocrine and Metabolic Diseases of the National Health Commission of the PR China, Shanghai Key Laboratory for Endocrine Tumor, State Key Laboratory of Medical Genomics, Ruijin Hospital, Shanghai, China

**Keywords:** primary hyperparathyroidism, clinical phenotype, famine, adult exposure, early life exposure

## Abstract

**Background & Aims:**

Primary hyperparathyroidism(PHPT) has been evolving into a milder asymptomatic disease. No study has assessed the association between famine exposure and such a shift. We aim to explore the effects of China’s Great Famine exposure on the changing pattern of PHPT phenotypes.

**Methods:**

750 PHPT patients diagnosed from 2000 to 2019 were studied. The clinical presentations were compared between them in recent 10 years (2010-2019) and previous 10 years (2000-2009). Participants were then categorized into fetal, childhood, adolescent, adult exposure, and unexposed groups. Logistic regression was used to estimate the odds ratios (ORs) and confidence intervals (CIs) of famine exposure as factors contributing to the changes in the clinical presentations of PHPT.

**Results:**

Serum levels of PTH, albumin-corrected Ca, tumor size, eGFR, BMDs (all P<0.001), and clinical symptoms became milder in recent 10 years. Famine exposure (72.6% vs 58.4%, P<0.001), especially the adult exposure (18.8% vs 4.1%, P<0.001)was significant less in recent 10 years. The ORs (95%CIs) of having upper 3^rd^ tertile PTH were 2.79(1.34,5.8), 2.07(1.04,4.11), 3.10(1.15,8.38) and 8.85(2.56,30.56) for patients with fetal, childhood, adolescent and adult famine exposure, respectively. The ORs (95%CIs) of upper 3^rd^ tertile albumin-corrected Ca and upper 3^rd^ tertile of tumor size was 4.78(1.39, 16.38) and 4.07(1.12,14.84) for participants with adult famine exposure, respectively. All these associations were independent of age, sex, disease duration and other confounders.

**Conclusions:**

The clinical manifestations of PHPT in China continue to be milder. Exposure to famine is associated with PHPT. Less famine exposure might be responsible for the mile form of PHPT in recent years.

## Introduction

Primary hyperparathyroidism (PHPT) is the third largest endocrine disease in the world with the main features of hyperparathyroidism and hypercalcemia (1, 2). The classical symptoms of PHPT involve bone, kidney, gastrointestinal manifestations, such as bone pain, fracture, osteoporosis, urolithiasis, nausea, vomiting, and etc. There are also some non-classical features, including neuropsychiatric, cardiovascular and metabolic abnormities (3).

Since 1970s, the clinical presentations of PHPT have changed around the world, especially in most Western countries and some developing countries in Asia, but not in Africa (4), from a symptomatic disorder to a mild or asymptomatic one (5–9). It was generally accepted that such a change is due to the widely routine measurement of electrolytes including serum calcium levels with automatic biochemical analyzers and neck ultrasonography which incidentally identifies parathyroid lesion(s) (8, 10). However, it is still of interest to ask whether there is any other factor responsible for such a change. Why the mild cases are mostly observed in developed Western countries, and in those developing countries with better social-economic condition, could it be related with nutritional status?

In fact, previous studies have already shown that nutritional status has a profound impact on many diseases. Numerous studies provided evidence that long-term malnutrition, such as famine, is closely related to chronic and metabolic diseases, such as cardiovascular diseases (11), diabetes (12, 13), non-alcoholic fatty liver disease (14),thyroid disorders (15) and osteoporosis (16). It is noteworthy that most such studies investigated the impacts of famine exposure in early life (fetal, childhood, adolescence exposure) on the risk of adulthood diseases, a few is reported on the impact of famine exposure during adulthood (17, 18).

The China’s Great Famine occurred in 1959-1962 (19), which was the world’s largest famine, it provides a unique opportunity to explore the effects of nutritional status on human health and diseases. Therefore, using the data collected from Jan, 2000-Dec, 2019 of PHPT patients in our center, we aimed to explore the changing clinical pattern of PHPT, and most importantly, the impact of famine exposure at different stages of life (fetal, childhood, adolescence and adult) on the changes of clinical phenotypes of PHPT in 20 years. We hypothesized that famine exposure in early and adult life is associated with the biochemical and clinical severity of PHPT.

## Materials and Methods

From Jan, 2000 to Dec, 2019, there were 810 cases of PHPT patients in Department of Endocrine and Metabolic Diseases, Ruijin Hospital, Shanghai Jiao Tong University School of Medicine. However, patients with parathyroid carcinoma (N=60) were excluded. For those with secondary hyperparathyroidism due to vitamin D deficiency, they were managed in the out-patient department; for those with secondary and tertiary hyperparathyroidism caused by renal failure, they were handled by doctors from the Department of Nephrology. Multiple endocrine neoplasia is caused by genetic abnormalities which are pathologically different from sporadic PHPT, and thus is beyond the purpose of the current study. Therefore, these patients were also not included in the current study.

A total of 750 patients with PHPT in our center were enrolled in this study. The diagnosis of parathyroid adenoma was established based on histopathology and immunohistochemistry. A mass was considered an adenoma if it fulfilled all of the following three criteria: 1) absence of fat cells within the mass; 2) absence of a lobular pattern; 3)presence of a well-defined demarcation between it and the surrounding parathyroid tissue with no demonstrable blending between the two (20).

In the current study, we included PHPT patients who were born between 1921 and 1995, and collected their baseline data. Participants were categorized into four exposure-age cohorts that defined as famine exposure in different periods of life: fetal exposure (born between 1959 and 1961, N=78), childhood exposure (exposed at ages 0-9 years, born between 1949 and 1959, N=239), adolescent exposure (exposed at ages 10-17 years, born between 1941 and 1949, N=90), adulthood exposure (exposed at ages over18 years, bore before 1941, N=58) and unexposed (born after 1962, N=285).

This study was approved by the Medical Ethics Committee at the Institutional Review Board of Ruijin Hospital, Shanghai Jiao Tong University School of Medicine.

### Data Collection

All the information pertaining to demographic characteristics, symptoms of PHPT (osteoporosis, bone pain, fracture history, urolithiasis, gastritis and gastric ulcer, nausea and vomiting) and disease duration were recorded. Height was measured to the nearest 0.1 cm, and weight was recorded to the nearest 0.1 kg with the participant wearing light clothing. BMI was calculated as weight in kilograms divided by height in meters squared.

All participants were required to fast for at least 10 hours before blood samples were collected to precede biochemical evaluation. The biochemical blood parameters that were tested included serum concentrations of calcium (Ca), phosphorus (P), albumin (Alb), parathyroid hormone (PTH), 25-hydroxyvitamin D [25(OH)D], creatinine-based glomerular filtration rate (GFR) as estimated GFR (eGFR), 24-hour urinary calcium (24hUCa) and phosphorus (24hUP) excretion. Serum Ca and P were measured using an automatic biochemical analyzer (Modular E170, Roche, Basel, Switzerland). Serum level of PTH was measured by intact immunoradiometric assay (ARCHITECT i2000sr, Abbott, Chicago, IL). The serum 25(OH)D concentration was measured with an electrochemiluminescence immunoassay (Roche Cobas 601)[Vitamin D3(25-OH) kit before 2011, and after that, Vitamin D total kit]. Fasting plasma glucose (FPG) was tested by the glucose oxidase method on an autoanalyzer (Modular P800; Roche, Basel, Switzerland). The albumin-corrected serum calcium level (mmol/L) was calculated using the following formula: albumin-corrected Ca(mmol/L) = measured total serum calcium(mmol/L) + [0.02 × (40 - serum albumin concentration(g/L)]. The formula for the calculation of eGFR is eGFR(ml/(min*1.73m^2^)=186×(Scr)^-1.154^×(age)^-0.203^×(0.742 if Female) (Scr, Serum creatinine).

Bone mineral densities (BMDs) at lumbar spine 1-4 (L1-L4), femoral neck (FN) and total hip (TH) were measured using a dual-energy X-ray absorptiometer (DXA)(Lunar Prodigy; GE Medical Systems, Madison, Wisconsin). Complete DXA data were available for 511 patients. The disease duration was recorded based on the first recognition of symptom(s) related to hyperparathyroidism in symptomatic patients or the first abnormal serum calcium or PTH level discovered by laboratory tests or parathyroid lesion found by neck ultrasonography in asymptomatic patients.

### Statistical Analysis

All statistical analyses were carried out with R 4.0.0 software (R Foundation, Vienna, Austria). The Shapiro-Wilk test was used to test whether continuous variables are normally distributed. Continuous variables were represented using the mean ± SD or mean ± SEM as otherwise specified for normally distributed variables and medians with 25th and 75th percentiles for non-normally distributed variables. Categorical variables were represented by frequency (percentage). For normal distribution data, the comparison between the two groups used the independent sample t test, the comparison between more than two groups used ANOVA, and the multiple comparison used Tukey’s fixed gap test (Tukey HSD); if the data was skewed, the Wilcoxon rank sum test was used for comparison, Kruskal-Wallis rank sum test was used for comparison between more than two groups, Holm’s method was used for multiple comparisons; chi-square test was used for comparison between categorical variables groups. Covariance analysis was used to correct confounders between groups. Spearman correlation was used to test the correlation between target variables and related variables. Odds ratios (ORs) and 95% confidence intervals (CIs) were calculated by using multivariable adjusted logistic regression model to analyze the associations of famine exposure with PHPT clinical phenotype. The accepted level of statistical significance was p<0.05.

## Results

### Demographic, Clinical Phenotypes and Famine Exposure in PHPT Patients From 2000-2019

750 patients with PHPT were divided into two groups: group A (187 patients diagnosed from 2000 to 2009) and group B (563 patients diagnosed from 2010 to 2019). The study included 540 females (72.0%) and 210 males (28.0%) with a sex-ratio of female to male 2.57:1. The clinical characteristics of two groups are summarized in **Table 1**.

**Table 1:** Changes over time in demographic, clinical findings and famine exposure in patients with PHPT from 2000-2019.

	2000-2009 (group A)	2010-2019 (group B)	P-value
Number of cases	187	563	
Sex (Female %)	118 (63.4%)	421 (74.8%)	**0.004**
Age (years)	52.0 [42.0, 59.8]	55.0 [46.0, 63.0]	**0.035**
Age at onset (years)	50.6 [36.0, 58.3]	51.9 [41.9, 60.0]	0.127
Disease duration (years)	1.75 [0.23, 4.00]	1.00 [0.25, 4.00]	0.540
BMI (kg/m2)	21.9 [20.5, 24.4]	22.9 [20.8, 25.3]	0.090
Serum PTH (pg/mL)	423 [213, 1170]	213 [138, 394]	**<0.001**
Albumin-corrected serum calcium (mmol/L)	2.95 [2.73, 3.30]	2.73 [2.57, 2.92]	**<0.001**
Serum phosphate (mmol/L)	0.766 ± 0.192	0.883 ± 0.196	**<0.001**
Serum 25(OH)D (nmol/L)	33.5 [23.3, 45.1]	28.9 [20.7, 40.7]	**0.002**
Urinary calcium (mmol/24h)	6.63 [5.02, 10.1]	6.25 [4.42, 8.37]	**0.018**
Alkaline phosphatase (IU/L)	123 [83.0, 229]	92.0 [67.0, 129]	**<0.001**
Tumor size (cm)	2.50 [2.00, 3.20]	1.70 [1.20, 2.45]	**<0.001**
BMD (g/cm2)			
L1-L4	0.911 ± 0.208	0.947 ± 0.196	0.129
Femoral Neck	0.725 ± 0.159	0.770 ± 0.150	**0.016**
Total Hip	0.757 ± 0.183	0.801 ± 0.155	**0.033**
eGFR	71.5 [48.5, 97.0]	98.7 [81.9, 110]	**<0.001**
Osteoporosis/Osteopenia	95 (84.1%)	281 (73.4%)	**0.027**
Fracture	31 (17.3%)	60 (10.7%)	**0.027**
Bone pain	83 (46.4%)	154 (27.4%)	**<0.001**
Urolithiasis	93 (52.0%)	243 (43.2%)	0.051
Nausea and vomiting	28 (15.6%)	61 (10.8%)	0.088
Exposure	135 (72.6%)	329 (58.4%)	**<0.001**
Fetal exposure	13 (7.0%)	64 (11.4%)	0.096
Childhood exposure	63 (33.9%)	176 (31.3%)	0.526
Adolescent exposure	24 (12.9%)	66 (11.7%)	0.697
Adult exposure	35 (18.8%)	23 (4.1%)	**<0.001**
asymptomatic PHPT	34 (18.3%)	207 (36.8%)	**<0.001**

Data are expressed as the mean ± SD, median value [interquartile range] or as n (%), as appropriate. Significant associations are indicated in bold.

P < 0.05 was considered statistically significant.

PTH, parathyroid hormone; 25(OH)D, 25-hydroxyvitamin D; BMD, Bone mineral density; eGFR, estimated glomerular filtration rate.

The mean age of the whole group was 54.0[46.0,62.0] years. Patients in group B had an older age than those in group A (P=0.035). There was no difference in the age at onset and disease duration between two groups. Comparing previous 10 years, there were significant decreases in serum levels of PTH, albumin corrected-Ca and alkaline phosphatase (ALP), as well as tumor size over time (all P<0.001), whilst serum P concentration (P<0.001), eGFR (P<0.001) and BMDs at FN(P=0.016) and TH (P=0.033) increased in recent 10 years.

Changes of clinical manifestations over time included a decrease in the rate of bone and digestive system involvement (**Table 1**). Compare with group A, osteoporosis/osteopenia was significantly less frequent in group B (73.4% vs. 84.1%; P=0.027). As to fracture, 91 (12.1%) subjects in total developed fragility fractures previously. The declining percentages of fracture were noticed between the two groups (17.3% in group A and 10.7% in group B, P=0.027). The proportion of bone pain witnessed a sharp drop (46.4% in group A vs 27.4% in group B, P<0.001). Nausea and vomiting showed a non-significant decreased incidence in group B (10.8%) as compared with group A(15.6%)(P=0.088). 336 PHPT patients (44.8%) developed urolithiasis at presentation with a marginal significant decrease in recent 10 years (43.2% vs 52.0%, P=0.051). There was an improvement in eGFR over time (P<0.001). The percentages of asymptomatic PHPT were 18.3% in group A (2000-2009) and 36.8% in group B (2010-2019)(P<0.001),respectively.

Also, as shown in **Table 1**, compared with previous 10 years, the famine exposure in recent 10 years showed a significant decrease from 72.6% to 58.4% (P<0.001), especially the adult exposure from 18.8% to 4.1% (P<0.001).

### The Relationship Between Famine Exposure and Clinical Phenotypes of PHPT

As shown in **Table 2**, compared with the unexposed group, after adjustment for age and sex, serum levels of PTH, albumin-corrected Ca, and ALP in the childhood exposure, adolescent exposure and adult exposure group were significantly increased, while BMDs at L1-L4, FN and TH in these exposure groups were significantly reduced. PHPT patients with fetal exposure also had higher circulating PTH level than unexposed ones. PHPT patients with famine exposure also had more clinical symptoms, such as osteoporosis/osteopenia, fracture history, bone pain, nausea and vomiting, except for urolithiasis. But there was no difference of the percentages of asymptomatic PHPT in famine exposure at different stages of life.

**Table 2:** The clinical data of primary hyperparathyroidism (PHPT) patients in famine exposure at different stages of life.

	Unexposed (Ref)	Fetal exposure	Childhood exposure	Adolescent exposure	Adult exposure	P-value	F vs Ref	C vs Ref	Ado vs Ref	Adu vs Ref
Number of cases	285	78	239	90	58					
Sex (Female %)	188 (66.0%)	61 (78.2%)	183 (76.6%)	72 (80.0%)	36 (62.1%)	**0.005**				
Age (years)	41.0 [33.0, 48.0]	53.0 [49.0, 55.8]	58.0 [55.0, 62.0]	67.5 [64.0, 70.0]	75.0 [71.0, 78.0]	**<0.001**	**<0.001**	**<0.001**	**<0.001**	**<0.001**
Age at onset (years)	38.0 [30.4, 45.9]	51.0 [47.8, 54.2]	56.0 [51.9, 60.0]	64.7 [60.3, 68.0]	72.5 [68.9, 77.0]	**<0.001**				
Disease duration (years)	1.00 [0.170, 4.00]	1.00 [0.250, 4.08]	1.00 [0.170, 4.00]	1.75 [0.417, 5.00]	1.00 [0.170, 3.25]	0.294				
BMI (kg/m2)	23.57 ± 0.33	23.48 ± 0.41	22.81 ± 0.26	21.81 ± 0.49	21.18 ± 0.70	**0.035**				0.056
Serum PTH (pg/mL)	239.9 ± 51.4	482.2 ± 66.3	502.4 ± 40.5	703.9 ± 72.6	1112.9 ± 99.7	**<0.001**	**0.027**	**0.004**	**<0.001**	**<0.001**
Albumin-corrected serum calcium(mmol/L)	2.72 ± 0.03	2.84 ± 0.04	2.86 ± 0.03	2.94 ± 0.05	3.20 ± 0.06	**<0.001**		**0.017**	**0.010**	**<0.001**
Serum phosphate (mmol/L)	0.893 ± 0.018	0.840 ± 0.023	0.855 ± 0.014	0.806 ± 0.025	0.760 ± 0.034	**0.032**				**0.027**
Serum 25(OH)D (nmol/L)	35.41 ± 1.76	36.16 ± 2.25	32.80 ± 1.38	26.85 ± 2.69	31.66 ± 3.54	0.103				
Urinary calcium (mmol/24h)	6.87 ± 0.91	8.12 ± 1.21	8.34 ± 0.75	7.23 ± 1.36	9.11 ± 1.78	0.671				
Alkaline phosphatase (IU/L)	63.84 ± 27.23	173.53 ± 36.19	213.65 ± 21.78	332.01 ± 39.37	427.89 ± 55.03	**<0.001**		**0.001**	**<0.001**	**<0.001**
Tumor size (cm)	1.93 ± 0.10	2.24 ± 0.14	2.17 ± 0.09	2.24 ± 0.17	2.66 ± 0.23	0.134				
BMD(g/cm^2^)										
L1-L4	1.014 ± 0.020	0.949 ± 0.026	0.882 ± 0.018	0.868 ± 0.031	0.860 ± 0.044	**0.001**		**<0.001**	**0.008**	**0.049**
Femoral Neck	0.818 ± 0.016	0.780 ± 0.020	0.731 ± 0.014	0.671 ± 0.024	0.688 ± 0.033	**<0.001**		**0.002**	**<0.001**	**0.018**
Total Hip	0.854 ± 0.017	0.801 ± 0.021	0.765 ± 0.015	0.702 ± 0.025	0.691 ± 0.035	**<0.001**		**0.004**	**<0.001**	**0.002**
eGFR	98.36 ± 2.89	90.35 ± 3.88	90.25 ± 2.32	89.06 ± 4.18	76.54 ± 5.86	0.070				**0.033**
Osteoporosis/Osteopenia	121(61.1%)	39(70.9%)	122(85.9%)	61(93.8%)	34(91.9%)	**<0.001**		**<0.001**	**<0.001**	**0.002**
Fracture	21 (7.5%)	12 (15.4%)	32 (13.6%)	19 (21.6%)	7 (12.1%)	**0.007**			**0.004**	
Bone pain	72 (25.6%)	26 (33.3%)	77 (32.5%)	36 (40.9%)	26 (44.8%)	**0.012**			**0.045**	**0.045**
Urolithiasis	137 (48.1%)	43 (55.1%)	106 (44.4%)	32 (35.6%)	19 (32.8%)	**0.027**				
Nausea and vomiting	23 (8.2%)	8 (10.3%)	36 (15.2%)	10 (11.4%)	13 (22.4%)	**0.015**				**0.029**
asymptomatic PHPT	95 (33.3%)	20 (25.6%)	85 (35.6%)	28 (31.1%)	13 (22.4%)	0.236				

P values is adjusted for age and sex. Data are expressed as the mean ± SEM, median value [interquartile range] or as n (%), as appropriate. Significant associations are indicated in bold. P < 0.05 was considered statistically significant.

PTH, parathyroid hormone; 25(OH)D, 25-hydroxyvitamin D; BMD, Bone mineral density; eGFR, estimated glomerular filtration rate

For the PHPT related symptoms (**Table 3**), after adjusting age, gender, BMI, disease duration and eGFR, as compared with non-exposure group, the famine exposure was associated with a higher ORs of having osteoporosis/osteopenia, fracture, bone pain and urolithiasis. The ORs (95%CIs) of osteoporosis/osteopenia were 4.51 (1.97,10.31), 8.04 (2.05,31.49) and 5.71 (1.07,30.42) for participants with childhood (P<0.001), adolescent(P=0.003) and adult famine exposure (P=0.041), respectively. The ORs (95%CIs) of fracture were 3.25 (1.11,9.47), 3.7 (1.28,10.72) and 6.51 (1.53,27.75) for participants with fetal(P=0.031), childhood (P=0.016) and adolescent famine exposure (P=0.011), respectively. The ORs (95%CIs) of bone pain was 4.33 (1.27,14.81) for participants with adult famine exposure(P=0.020). The ORs (95%CIs) of urolithiasis was 1.83 (1.03,3.25) for participants with famine exposure(P=0.038). It was also revealed that famine exposure in childhood and adult was related with more digestive symptoms, such as nausea and vomiting after adjusting age and gender in model 2, however, this association was lost when disease duration and eGFR were further adjusted in model 3.

**Table 3:** ORs (95%CIs) for related symptoms in PHPT patients with famine exposure at different stages of life.

	Model 1 OR(95%CI)	P value	Model 2 OR(95%CI)	P value	Model 3 OR(95%CI)	P value
**Osteoporosis/osteopenia**						
Unexposed	1.0 (Ref.)		1.0 (Ref.)		1.0 (Ref.)	
Exposed	**3.79 (2.46,5.83)**	**<0.001**	**2.71 (1.42,5.18)**	**0.003**	**2.77 (1.36,5.64)**	**0.005**
Fetal exposure	1.55 (0.81,2.97)	0.184	1.64 (0.79,3.40)	0.183	1.54 (0.68,3.48)	0.301
Childhood exposure	**3.88 (2.23,6.74)**	**<0.001**	**4.19 (1.99,8.85)**	**<0.001**	**4.51 (1.97,10.31)**	**< 0.001**
Adolescent exposure	**9.70 (3.39,27.76)**	**<0.001**	**10.94 (3.01,39.79)**	**<0.001**	**8.04 (2.05,31.49)**	**0.003**
Adult exposure	**7.21 (2.14,24.30)**	**0.001**	**8.17 (1.73,38.61)**	**0.008**	**5.71 (1.07,30.42)**	**0.041**
**Fracture history**						
Unexposed	1.0 (Ref.)		1.0 (Ref.)		1.0 (Ref.)	
Exposed	**2.22 (1.33,3.71)**	**0.002**	**2.63 (1.26,5.51)**	**0.010**	**3.24 (1.31,7.99)**	**0.011**
Fetal exposure	**2.25 (1.05,4.81)**	**0.036**	**2.96 (1.22,7.19)**	**0.016**	**3.25 (1.11,9.47)**	**0.031**
Childhood exposure	**1.94 (1.09,3.47)**	**0.025**	**3.03 (1.26,7.29)**	**0.013**	**3.7 (1.28,10.72)**	**0.016**
Adolescent exposure	**3.41 (1.74,6.7)**	**<0.001**	**6.59 (2.04,21.31)**	**0.002**	**6.51 (1.53,27.75)**	**0.011**
Adult exposure	1.70 (0.69,4.21)	0.252	4.36 (0.96,19.76)	0.057	3.50 (0.47,25.96)	0.220
**Bone pain**						
Unexposed	1.0 (Ref.)		1.0 (Ref.)		1.0 (Ref.)	
Exposed	**1.62 (1.16,2.25)**	**0.004**	**1.71 (1.04,2.81)**	**0.033**	1.20 (0.67,2.14)	0.541
Fetal exposure	1.45 (0.84,2.49)	0.178	**1.93 (1.04,3.58)**	**0.037**	1.31 (0.63,2.72)	0.475
Childhood exposure	1.40 (0.95,2.05)	0.086	**2.24 (1.26,3.99)**	**0.006**	1.52 (0.78,2.97)	0.219
Adolescent exposure	**2.01 (1.22,3.32)**	**0.006**	**4.07 (1.81,9.12)**	**<0.001**	2.56 (0.99,6.59)	0.052
Adult exposure	**2.36 (1.32,4.22)**	**0.004**	**6.70 (2.46,18.3)**	**<0.001**	**4.33 (1.27,14.81)**	**0.020**
**Urolithiasis**						
Unexposed	1.0 (Ref.)		1.0 (Ref.)		1.0 (Ref.)	
Exposed	0.81 (0.60,1.08)	0.154	1.41 (0.89,2.24)	0.143	**1.83 (1.03,3.25)**	**0.038**
Fetal exposure	1.29 (0.78,2.14)	0.320	1.64 (0.93,2.89)	0.090	2.04 (1.40,4.18)	0.051
Childhood exposure	0.85 (0.60,1.20)	0.360	1.14(0.68,1.91)	0.628	1.53 (0.80,2.90)	0.195
Adolescent exposure	**0.60 (0.37,0.98)**	**0.043**	0.91 (0.43,1.93)	0.811	1.12 (0.43,2.89)	0.819
Adult exposure	**0.51 (0.28,0.93)**	**0.028**	0.75(0.29,1.93)	0.545	0.98 (0.28,3.38)	0.973
**Nausea and vomiting**						
Unexposed	1.0 (Ref.)		1.0 (Ref.)		1.0 (Ref.)	
Exposed	**1.91 (1.16,3.15)**	**0.011**	1.69 (0.82,3.47)	0.156	1.89 (0.81,4.45)	0.143
Fetal exposure	1.29 (0.55,3.00)	0.559	1.45 (0.56,3.75)	0.439	1.51 (0.52,4.42)	0.449
Childhood exposure	**2.02 (1.16,3.51)**	**0.013**	**2.45 (1.06,5.66)**	**0.036**	2.25 (0.85,5.94)	0.103
Adolescent exposure	1.44 (0.66,3.16)	0.359	1.92 (0.57,6.44)	0.289	1.57 (0.38,6.39)	0.531
Adult exposure	**3.25 (1.54,6.89)**	**0.002**	**4.86 (1.2,19.68)**	**0.027**	2.46 (0.44,13.86)	0.307

Model 1: unadjusted.

Model 2: adjusted for age, gender.

Model 3: adjusted for age, gender, BMI, disease duration, eGFR.

Bold values represent results with statistical significance.

### The Relationships Between Famine Exposure and the Severity of PHPT

To evaluate the association between famine exposure and severity of PHPT, patients were subgrouped according to the tertiles of serum PTH, albumin-corrected Ca levels and tumor size. As shown in **Table 4**, after adjustment for age, sex, BMI, disease duration and eGFR, the ORs (95%CIs) of having upper 3^rd^ tertile PTH were 2.79(1.34,5.8), 2.07(1.04,4.11), 3.10(1.15,8.38) and 8.85(2.56,30.56) for participants with fetal, childhood, adolescent and adult famine exposure, respectively. The ORs (95%CIs) of upper 3^rd^ tertile albumin-corrected Ca was 4.78(1.39, 16.38) for participants with adult famine exposure. The ORs (95%CIs) of upper 3^rd^ tertile of tumor size was 4.07(1.12,14.84) for participants with adult famine exposure.

**Table 4:** ORs (95%CIs) for upper 3^rd^ serum PTH, albumin-corrected calcium levels and tumor size in PHPT patients with famine exposure at different stages of life.

	Model 1OR (95%CI)	P value	Model 2OR (95%CI)	P value	Model 3OR (95%CI)	P value
**Upper 3rd PTH**						
Unexposed	1.0 (Ref.)		1.0 (Ref.)		1.0 (Ref.)	
Exposed	0.94 (0.68,1.29)	0.693	**1.81 (1.10,2.98)**	**0.020**	**1.85 (1.02,3.34)**	**0.042**
Fetal exposure	1.22 (0.71,2.08)	0.471	**2.56 (1.37,4.78)**	**0.003**	**2.79 (1.34,5.8)**	**0.006**
Childhood exposure	0.77 (0.52,1.12)	0.175	**2.21 (1.24,3.97)**	**0.008**	**2.07 (1.04,4.11)**	**0.038**
Adolescent exposure	0.88 (0.52,1.47)	0.617	**4.01 (1.77,9.06)**	**<0.001**	**3.10 (1.15,8.38)**	**0.026**
Adult exposure	1.54 (0.86,2.77)	0.145	**10.78 (3.92,29.66)**	**<0.001**	**8.85 (2.56,30.56)**	**<0.001**
**Upper 3rd Alb-Ca**						
Unexposed	1.0 (Ref.)		1.0 (Ref.)		1.0 (Ref.)	
Exposed	1.08 (0.79,1.48)	0.626	**1.64 (1.01,2.66)**	**0.048**	1.23 (0.68,2.23)	0.486
Fetal exposure	1.06 (0.62,1.81)	0.827	**2.05 (1.10,3.80)**	**0.023**	1.36 (0.65,2.86)	0.420
Childhood exposure	0.90 (0.62,1.30)	0.568	**2.26 (1.28,4.01)**	**0.005**	1.56 (0.79,3.08)	0.198
Adolescent exposure	1.10 (0.66,1.82)	0.719	**4.17 (1.87,9.31)**	**<0.001**	1.86 (0.69,5.00)	0.222
Adult exposure	**2.20 (1.23,3.91)**	**0.007**	**11.49 (4.24,31.11)**	**<0.001**	**4.78 (1.39,16.38)**	**0.013**
**Upper 3rd tumor size**						
Unexposed	1.0 (Ref.)		1.0 (Ref.)		1.0 (Ref.)	
Exposed	0.84 (0.61,1.18)	0.318	1.36 (0.83,2.24)	0.222	1.38 (0.78,2.42)	0.264
Fetal exposure	1.14 (0.64,2.03)	0.655	1.76 (0.93,3.33)	0.082	1.86 (0.90,3.83)	0.094
Childhood exposure	0.79 (0.54,1.15)	0.221	1.50 (0.86,2.61)	0.153	1.38 (0.74,2.60)	0.311
Adolescent exposure	**0.55 (0.32,0.95)**	**0.031**	1.45 (0.64,3.27)	0.373	1.24 (0.49,3.18)	0.649
Adult exposure	1.74 (0.79,3.82)	0.170	**5.90 (1.92,18.12)**	**0.002**	**4.07 (1.12,14.84)**	**0.033**

Model 1: unadjusted.

Model 2: adjusted for age, gender.

Model 3: adjusted for age, gender, BMI, disease duration, eGFR.

Bold values represent results with statistical significance.

## Discussion

The main findings of the study are: 1) The clinical and biochemical manifestations of PHPT in China are trending toward even milder in recent 10 years, and 2) both the early life and adult exposure to famine are associated the changing pattern of PHPT in this 20 years. To the best of our knowledge, this is the first study to evaluate the association between famine exposure and PHPT in adulthood.

In our previous study, we found that PHPT in China was evolving into a mild form from 2000 to 2010 (8). In the current study, such a changing patter continued. Comparing with the previous 10 years from 2000-2009, the biochemical features in PHPT patients, such as serum PTH, albumin-corrected Ca, ALP concentrations all became lower; and tumor size to be smaller; kidney function and BMDs turned to be better in recent 10 years from 2010-2019. Such a picture echoes the most recent findings from another hospital in our city (9) and other areas in Western countries.

Since the major purpose of this study is to evaluate whether famine exposure during early and adult life has anything related with PHPT, we then investigated the rate and type of exposures in different years. It was demonstrated that famine exposure, especially adult exposure was more pronounced in previous 10 years than recent 10 years. Detail analysis further revealed that even with the adjustment of age, gender, eGFR, and other confounders, our study revealed that exposure to famine conferred increased risk of severer PHPT, as evidenced by higher PTH, albumin-corrected Ca levels, lower BMDs, higher risk of osteoporosis/osteopenia, fracture and renal involvement. It was noteworthy that our results seemed to be more prominent in adult exposure group, while the findings from a recent meta-analysis involving 1.6 million subjects showing that early life exposure (fetal, childhood and adolescent) were more prone to develop diabetes as compared with adult exposure (21). The possible explanation for such a difference might be that adequate nutrition during the stage of growth and development is important for metabolic diseases (21), while for a tumor disease like PHPT, in addition to nutrition status, other factors may also play some role (see below for more discussion).

Bone is a continuous remodeling organ with critical bone development during adolescence, and peak bone mass obtained during early adulthood. Sufficient calcium, VitD and protein intake in early life, and even intrauterine or early postnatal life, is crucial for bone accretion (22). Experiencing malnutrition in these periods will compromise the genetic determined bone growth and maturation, leading to low bone mass, compromised bone micro-architecture as well as bone strength, and subsequently higher fracture risk (23). Such an explanation is supported by the evidence that famine exposure in early life is associated with lower BMDs, poorer bone quality as revealed by heal quantitative ultrasound (QUS), and higher risk of vertebral fracture as evidenced by a 2-cm height loss in adulthood (16). Even with the adjustment of calcium intake, age, and other factors, famine exposure in Hong Kong Chinese man and women is associated with decreased DXA measured BMD at FN and a 25% increased risk of osteoporosis (24).

In this study, we found that famine exposure is related with higher odds of being at upper 3rd tertile of serum level of PTH and albumin-corrected Ca, which are considered to be responsible for the symptoms and severity of PHPT. However, it is difficult to explain this phenomenon at mechanism level. In this study, PHPT patients with pathological confirmed adenoma were enrolled. The molecular pathogenesis of sporadic parathyroid adenomatosis is complex (25) not only involving mutations in CCND1/PRAD1, CDC73/HRPT2, MEN1, CaSR (25–27) and several other genes identified by the next-generation sequencing techniques (28) but also involving epigenetic alterations such as DNA methylation and histone modifications in sporadic parathyroid adenoma (29–31). Studies in etiology or mechanism of famine-related tumorigenesis and disease severity are not fully understood. The appropriate mechanism(s) linking genetic alteration, environmental changes and human health and disease might be epigenetics (18, 32) and two-hit hypothesis or multiple-hit model (33–35).

In a human study, it was demonstrated that prenatal famine exposure is associated with the hypomethylation of *IGF2* differentially methylated region (DMR) 60 years later (36), and disruption of IGF-2 gene expression may contribute to tumor progression and aggressive phenotype in MEN-1 (37). As a disease of tumor, it would be reasonable to hypothesize that in genetic predisposed individual, experiencing calories deprivation in early life and/or adult life as one hit, together with epigenetic modifications resulting from famine (18, 38), environmental insults related with famine, such as life style changes, alcohol drinking, tobacco smoking (38–41), and economic status (42), will induce cumulative and interactive responses (43) and culminate in the development of a disease or even a tumor. In addition, the accumulation of a series of gene alterations and epigenetic modifications as multiple hits, is not only responsible for the tumorigenesis but also the functionality of the tumor, the aggressiveness of the clinical course, and the recurrence of the tumor, even independent of histopathology score (39, 44–48). Although these findings are derived from other tumors, such as gastric cancer (39, 44, 45) and adrenocortical carcinoma (46–48), such hypothesis is still suggestive for this study and should be investigated in detail in clinical and basic researches of PHPT.

PHPT is also a disease of aberrant set-point for PTH secretion with the curve of inverse relationship between PTH release and calcium level shifts to the right (49). Previously, it was reported that prenatal nutrition deprivation may result in *in utero* resetting the hypothalamus-pituitary-adrenal axis by a so called fetal reprograming mechanism, and leading to overproduction of cortisol, however, this was not confirmed in a later experiment at least at adrenal level (50). Nevertheless, the role of famine in dysregulation of set-point of PTH secretion could also be investigated.

It could be argued that in this study, age rather than famine itself is a contributing factor. It is expected that age was definitely different among various exposure groups from fetal to adult exposure. It was reported that in PHPT, serum calcium level is more closely related with cell number, tumor volume and serum PTH concentration, while age and sex are not contributing factors (51). In our analysis, age and disease duration were adjusted, and the results were still supportive the relationship between famine exposure and PHPT biochemical as well as clinical manifestations.

If the famine exposure is finally proved to be true to explain the occurrence and severity of PHPT, then it could be used to explain the changing pattern of PHPT in this study. In our cohort, significantly less proportion of PHPT patients in recent 10 years experienced famine than that of previously 10 years. The fact that there was a more than 19.6% decrease of exposed group in recent 10 years from 72.6% to 58.4%, and 78.2% relative reduction in adult exposure from 18.8% to 4.1%, which accounted for most of the severer clinical and laboratory findings of PHPT independent of age, disease duration, eGFR and other confounders should not be neglected and might be a factor responsible for the continuous milder form of PHPT in recent 10 years.

Our study has some limitations. First, we did not perform an analysis on the effects of famine severity, duration and geographical distribution on the phenotypes and severity of PHPT, we did not record the regions where our patients were living during the famine period. Second, we could not distinguish fetal exposed from infant exposed group, since some individuals experienced famine during both fetal period and infancy. Third, famine length might not be consistent across all included patients, which may influence the stability of our results. Fourth, as mentioned in the Method, the methodologies for the measurement of serum 25(OH)D concentration in the two periods were different and thus not comparable. Using more accurate method, such as Liquid Chromatography (LC)-Mass Spectroscopy (MS)/MS, may reflect the real Vitamin D status in human bodies.

In summary, the results from the current study demonstrate that PHPT in Chinese patients are still changing and become milder. Exposure to China’ Great Famine during early life and adult life is associated with clinical presentations of PHPT, and might be another explanation for the milder form of PHPT observed in recent years in Chinese patients.

## Data Availability Statement

The raw data supporting the conclusions of this article will be made available by the authors, without undue reservation.

## Ethics Statement

The studies involving human participants were reviewed and approved by Ruijin Hospital Ethics Committee. Written informed consent for participation was not required for this study in accordance with the national legislation and the institutional requirements.

## Author Contributions

Study design – J-mL, BT, and T-jY. Data extraction and analysis – T-jY. Writing of the manuscript – All authors. Critical review – J-mL, BT and L-hS. Final approval – All authors.

## Conflict of Interest

The authors declare that the research was conducted in the absence of any commercial or financial relationships that could be construed as a potential conflict of interest.

## Publisher’s Note

All claims expressed in this article are solely those of the authors and do not necessarily represent those of their affiliated organizations, or those of the publisher, the editors and the reviewers. Any product that may be evaluated in this article, or claim that may be made by its manufacturer, is not guaranteed or endorsed by the publisher.
